# Creeping eruptions: cutaneous larva migrans

**DOI:** 10.3402/jchimp.v3i3-4.21833

**Published:** 2013-12-17

**Authors:** Suzanne J. Supplee, Shobhit Gupta, Richard Alweis

**Affiliations:** 1 Department of Internal Medicine, The Reading Health System, West Reading, PA, USA; 2 Department of Medicine, Jefferson Medical College, Philadelphia, PA, USA

**Fig. 1:**
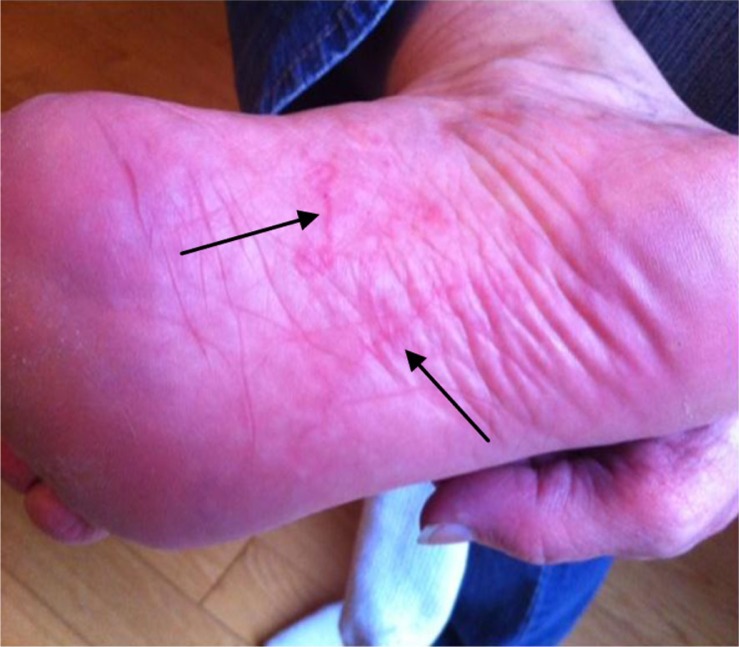
Serpiginous, erythematous lesions.

A 53-year-old woman developed an intensely pruritic rash on the plantar aspect of her right foot within 1 week of returning from a vacation in Jamaica, where she had walked barefoot on the beach (Fig. 1). The pattern revealed a classic serpiginous, elevated, erythematous lesion consistent with cutaneous larva migrans (CLM).

CLM is the most common travel-associated dermatological infection presented to primary care physicians (1). The initial manifestation of the rash may be a simple vesicle, which can lead to an initial misdiagnosis. Due to unfamiliarity with the rash, as well as the variable initial presentation, it is estimated that the initial diagnosis is incorrect in 55% of cases (2). The lesions are caused by a hookworm from the intestines of dogs, cats, or other mammals, most commonly *Acylostoma braziliense* and *Ancylostoma caninum* species (3). Vacationers to the topical beaches of the Caribbean, Africa, Asia, and South America usually recall stray cats and dogs occupying a beach they recently visited. The eggs are shed in the stool of these hosts and contaminate the soil or sand. When deposited on a moist surface, the eggs hatch and the larvae become infectious to the unsuspecting vacationer walking barefoot on the beach. Larvae penetrate the exposed skin surface and migrate through the epidermis. They track laterally, leaving a characteristic linear or serpentine, vesicular rash commonly referred to as ‘creeping eruptions’ (4). This is intensely pruritic in 98–100% of patients (1). Humans are accidental hosts, and the hookworm is unable to penetrate into the basal membrane, leading to death of the larvae.

Rare cases of Loffler's syndrome have been reported due to larval penetration of the lung causing pulmonary eosinophilia and a persistent cough (5). Although CLM due to hookworm infection is self-limited, irritating pruritus and potential for complications are reasons to accurately identify the disease and offer treatment. Laboratory testing is not felt to be helpful in making the diagnosis. First-line treatment is oral ivermectin or albendazole, usually requiring only one dose. If not resolved, subsequent doses may be given.

In summary, CLM is an easily missed clinical diagnosis requiring a thorough travel history and recognition of the cutaneous lesions, that is, the classic pruritic serpiginous rash.
